# Biomechanical and metabolic aspects of backward (and forward) running on uphill gradients: another clue towards an almost inelastic rebound

**DOI:** 10.1007/s00421-020-04474-7

**Published:** 2020-08-25

**Authors:** L. Rasica, S. Porcelli, A. E. Minetti, G. Pavei

**Affiliations:** 1 Institute of Biomedical Technologies, National Research Council, Segrate, Italy; 2 Department of Molecular Medicine, University of Pavia, Pavia, Italy; 3 Laboratory of Physiomechanics of Locomotion, Department of Pathophysiology and Transplantation, Physiology Division, University of Milan, Via Mangiagalli 32, 20133 Milan, Italy

**Keywords:** Backward acceleration, Efficiency, Mechanical work, Metabolic cost, Metabolic power

## Abstract

**Purpose:**

On level, the metabolic cost (*C*) of backward running is higher than forward running probably due to a lower elastic energy recoil. On positive gradient, the ability to store and release elastic energy is impaired in forward running. We studied running on level and on gradient to test the hypothesis that the higher metabolic cost and lower efficiency in backward than forward running was due to the impairment in the elastic energy utilisation.

**Methods:**

Eight subjects ran forward and backward on a treadmill on level and on gradient (from 0 to + 25%, with 5% step). The mechanical work, computed from kinematic data, *C* and efficiency (the ratio between total mechanical work and *C*) were calculated in each condition.

**Results:**

Backward running *C* was higher than forward running at each condition (on average + 35%) and increased linearly with gradient. Total mechanical work was higher in forward running only at the steepest gradients, thus efficiency was lower in backward running at each gradient.

**Conclusion:**

Efficiency decreased by increasing gradient in both running modalities highlighting the impairment in the elastic contribution on positive gradient. The lower efficiency values calculated in backward running in all conditions pointed out that backward running was performed with an almost inelastic rebound; thus, muscles performed most of the mechanical work with a high metabolic cost. These new backward running *C* data permit, by applying the recently introduced ‘equivalent slope’ concept for running acceleration, to obtain the predictive equation of metabolic power during level backward running acceleration.

## Introduction

Backward running is commonly used in rehabilitation and as an injury prevention strategy (e.g. Soligard et al. 2008; Gilchrist et al. 2008; Heiderscheit et al. 2010; Rössler et al. 2016), thanks to the reduced knee joint forces and lower vertical peak of the ground reaction force compared with forward running (Flynn and Soutas-Little 1995; Sussman et al. 2000; Roos et al. 2012). Moreover, the reverse direction of the movement gives the possibility to involve and train different muscles groups (DeVita and Stribling 1991; Flynn and Soutas-Little 1995; Sterzing et al. 2016); for a comprehensive review on backward running see Uthoff et al. (2018). An increasing number of backward running competitions have also been organised all over the world (also the RetroRunning world championship), with athletes training specifically backward for improving their performance.

On level, the metabolic demand of backward running is higher than forward running (Reilly and Bowen 1984; Flynn et al. 1994; Wright and Weyand 2001) probably due to a higher muscle activation (Flynn and Soutas-Little 1993, 1995; Wright and Weyand 2001; Sterzing et al. 2016) and/or a reduced elastic energy utilisation (Cavagna et al. 2011, 2012). This lower elastic contribution could be caused by the inverse approach of the foot on the ground that does not allow to store and recoil the energy from Achilles tendon or foot arch. Up to now, on level, no studies have analysed the mechanical work and metabolic cost of backward running concurrently so that conclusions about efficiency and elastic energy were inferred only indirectly.

When moving on positive gradient, the energy saving mechanism of forward running is impaired (Minetti et al. 1994). When running uphill the downward trajectory of the body centre of mass is reduced and less energy can be stored in the elastic elements of the lower limbs, which decreases the overall running efficiency (Minetti et al. 1994). There are no studies on the metabolic aspects (or efficiency) of backward running on gradient yet. However, it has been shown that the difference in metabolic cost between forward and backward walking was 100% on level, and decreased to 5–8% at gradients steeper than + 15% (Minetti and Ardigò 2001) and this decrement was addressed to the impairment in the pendulum like motion while walking uphill.

Based on this general knowledge, the analysis of mechanical and metabolic aspects of backward running on gradient would test the hypothesis of the higher metabolic cost and the possible decreased efficiency in backward than forward running due to the impairment in the elastic energy utilisation.

## Materials and methods

### Subjects

Eight male endurance runners (age: 25.6 ± 3.2 year, height: 1.76 ± 0.07 m, mass: 68.4 ± 6.6 kg, $\dot{V}$ O_2max_: 65.7 ± 6.2 mlO_2_ kg^−1^ min^−1^; mean ± SD) took part in the study. Each subject was fully informed about the aims, methods, and risks associated with participation and gave his written informed consent before the start of the study. All procedures were in accordance with the Declaration of Helsinki and the study was approved by the local ethics committee. Subjects undertook three familiarisation sessions with backward running at all speeds and gradients to get used with balance and proprioception while moving backward. After familiarisation, subjects came to the laboratory six times to complete the entire protocol.

### Experimental protocol

Subjects visited the laboratory on six different not-consecutive days. This protocol was designed to avoid any fatigue effect due to the high metabolic and neuromuscular demand of each acquisition; the comparison between forward and backward running on the same subject was performed to avoid any mechanical or metabolic confounding factors; a number of speeds were tested to check the metabolic cost behaviour. On day 1, subjects ran forward on level at 2.78 m s^−1^, on gradient + 5% at 2.5 m s^−1^ and + 10% at 2.22 m s^−1^, with 15 min of recovery among trials. On day 2, subjects ran forward on gradient + 15% at 1.94 m s^−1^ and + 20% at 1.67 m s^−1^, with 15 min of recovery between trials. On day 3, subjects ran backward on level at 1.67 m s^−1^, on gradient + 5% at 1.53 m s^−1^ and + 20% at 1.11 m s^−1^, with 15 min of recovery among trials. On day 4, subjects ran backward on gradient + 10% at 1.11 m s^−1^, 1.39 m s^−1^ and 1.67 m s^−1^, with 15 min of recovery among trials. On day 5, subjects ran backward on gradient + 15% at 1.11 m s^−1^, 1.25 m s^−1^, 1.39 m s^−1^ and 1.67 m s^−1^, with 15 min of recovery among trials. All acquisitions lasted 5 min. On day 6, kinematics data for all conditions were recorded (see below). The mechanical parameters (and efficiency) were compared between backward and forward running at each slope pairwise at these speeds: 1.67, 1.53, 1.39, 1.25, 1.11, 0.97 m s^−1^ for backward running and 2.78, 2.50, 2.22, 1.94, 1.67, 1.39 m s^−1^ for forward running at 0, + 5, + 10, + 15, + 20 and + 25% gradient, respectively.

### Metabolic measurements

Each experimental session was preceded by an 8-min stand resting oxygen consumption ($\dot{V}$O_2_, mlO_2_ kg^−1^ min^−1^) assessment after which subjects started running on the treadmill. Data acquisition lasted 5 min in order to reach a steady state $\dot{V}$O_2_. Pulmonary ventilation, oxygen consumption and carbon dioxide production were analysed breath by breath by a metabolic cart (Vmax229, SensorMedics, The Netherlands). The metabolic cost of running (C, J kg^−1^ m^−1^, Margaria et al. 1963) was calculated from the data collected during the last minute of exercise by dividing the measured net $\dot{V}$O_2_ (total – resting $\dot{V}$O_2_) by the running speed. The unit conversion from mlO_2_ to metabolic J was achieved by considering the mean respiratory exchange ratio ($\dot{V}$CO_2_ $\dot{V}$O_2_^−1^) for each acquisition. At rest and during recovery (3rd and 5th minute) 20 μL of capillary blood was obtained from a preheated earlobe for the determination of blood lactate concentration ([La^−^]_b_) by an enzymatic method (Biosen 5030, EKF, Germany).

### Kinematics

Three-dimensional (3D) body motion was collected by an 8-camera system (6 Vicon MX 1.3, 2 T20-S, Oxford Metrics, UK), by sampling at 100 Hz the spatial coordinates of 18 reflective markers located on the main joint centres (Minetti et al. 1993; Pavei et al. 2017), while the subject was running on a treadmill (Ergo LG Woodway, Germany). Marker positions were filtered through a ‘zero-lag’ second-order Butterworth low pass filter with a cutoff frequency detected by a residual analysis on each marker coordinate (Winter 1979). Each acquisition lasted 1 min and the time course of the 3D body centre of mass (BCoM) position was computed from an 11-segment model (Minetti et al. 1993; Pavei et al. 2017) based on Dempster inertial parameters of body segments (Winter 1979). From the BCoM 3D trajectory, the time course of potential (PE) and kinetic (KE) energies was computed to obtain the total mechanical energy (TE = PE + KE). The summation of all increases in TE time course constitutes the positive external work (*W*_EXT_, J kg^−1^ m^−1^), the work done to accelerate and lift the BCoM (Cavagna et al. 1963; Cavagna et al. 1976). The work necessary to rotate and accelerate limbs with respect to BCoM (*W*_INT_, J kg^−1^ m^−1^) (Cavagna and Kaneko 1977; Willems et al. 1995) was also calculated (according to Minetti et al. 1993) and summed to *W*_EXT_ to obtain the total mechanical work (*W*_TOT_, J kg^−1^ m^−1^). The frictional component of *W*_INT_ (Minetti et al 2020) was not included in the present calculation. The negative external work (*W*_EXT_^−^, J kg^−1^ m^−1^), the decreases in TE time course, was analysed as percentage of ‘comprehensive’ external mechanical work (= (*W*_EXT_) + (*W*_EXT_^−^)) in gradient locomotion, as suggested by Minetti et al. (1994). The ratio between *W*_TOT_ and *C* was used to estimate locomotion efficiency. Elastic energy contribution was estimated at each step as the difference between the mechanical equivalent of *C* and *W*_TOT_. *C* was converted into *W*_TOT_ by multiplying by an efficiency value of the positive work of 0.28 (Woledge et al. (1985) reported a range of 0.25–0.30 for positive work muscle efficiency), then the measured *W*_TOT_ was subtracted from it. The result, multiplied by the progression speed and divided by step frequency, provides an estimate of the elastic energy stored in a step. The elastic energy value of forward running on level was set to 1, and all the other conditions are reported as (sub)multiples. All data were analysed with custom-written Labview programs (release 10, National Instruments, USA).

### Statistics

Data were presented as mean ± SD and compared between running conditions using paired *t* test; difference among speeds were compared using one-way ANOVA for repeated measures and Bonferroni post hoc test; significance level was set at *p* < 0.05. Statistical analyses were performed with SPSS version 20 (IBM).

## Results

### Metabolic cost

**Fig. 1:**
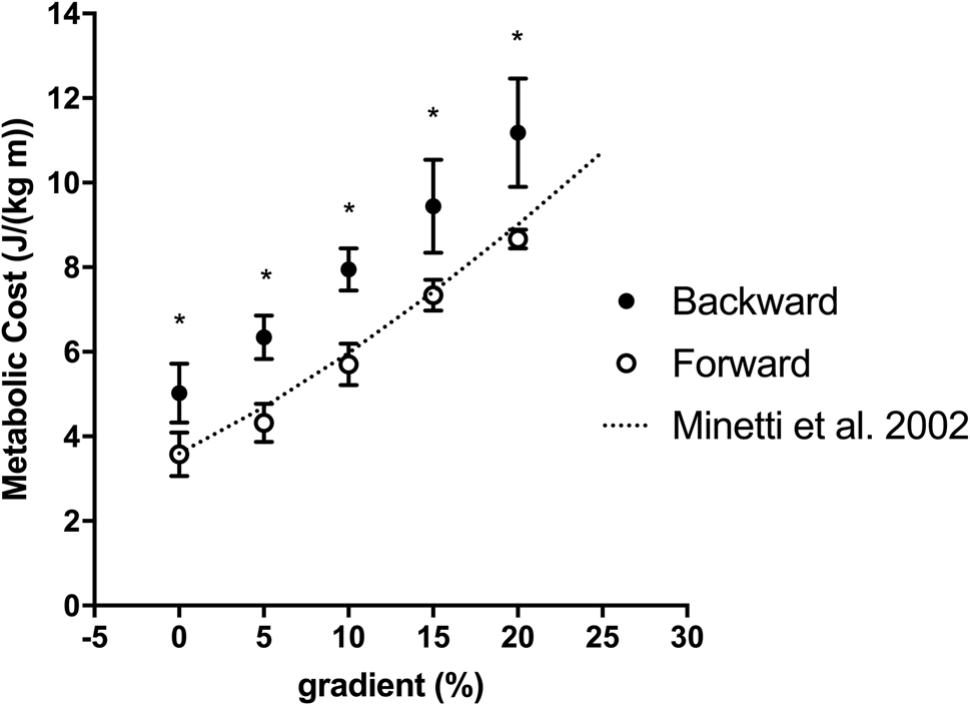
Metabolic cost (J kg^−1^ m^−1^) as a function of gradient (%). Black circles represent backward running, and white circles represent forward running. The superimposed dotted line represents the Minetti et al. 2002 equation of metabolic cost on gradient and well fit the experimental data. Backward running cost is always higher than forward running (*p < 0.01) on average of 35%. Data are mean ± SD

Forward running *C* increased with slope and present data are comparable with Minetti et al. (2002) values (Fig. 1). Backward running *C* was significantly higher than forward running at each slope (*P* < 0.01, Fig. 1) and speed independent at the analysed gradients. Backward running *C* (J kg^−1^ m^−1^) can be computed as a function of gradient (with same units as in Fig. 1) with the equation: *C* = 0.31*gradient + 4.9 (*R*^2^ = 0.99). The difference between forward and backward running was almost constant among gradients 35 ± 7%.

### Biomechanical parameters

**Fig. 2:**
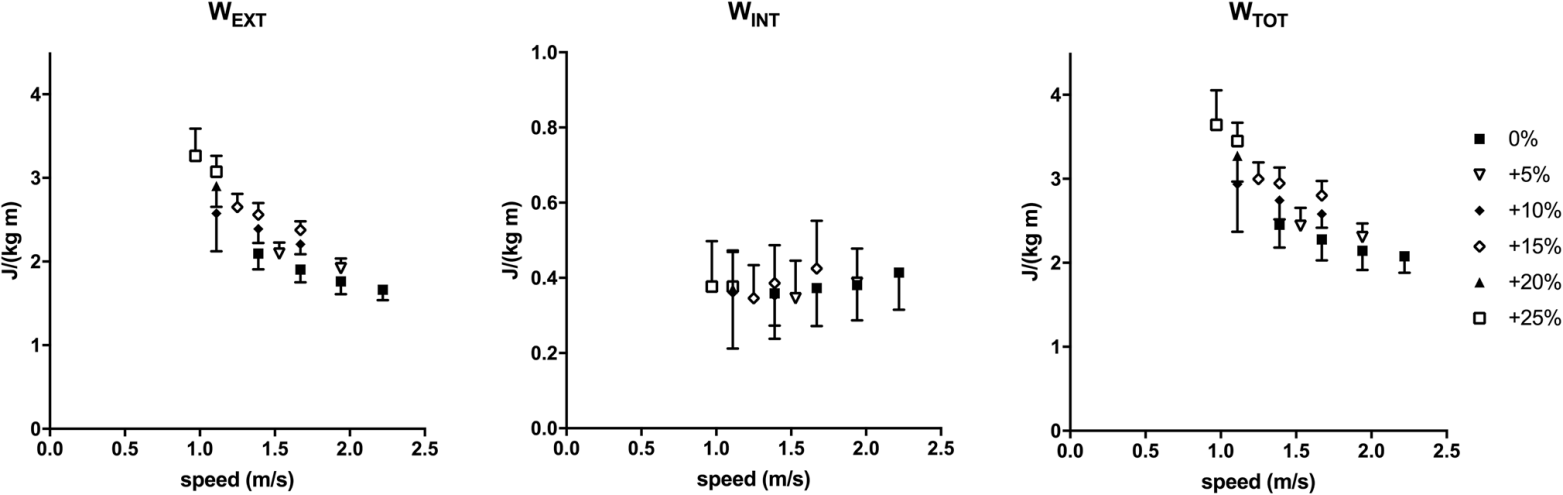
The mechanical external (*W*_EXT_), internal (*W*_INT_) and total (*W*_TOT_) work (J kg^−1^ m^−1^) as a function of speed (m s^−1^) in backward running is represented at the different investigated gradients. Data are mean ± SD

**Fig. 3:**
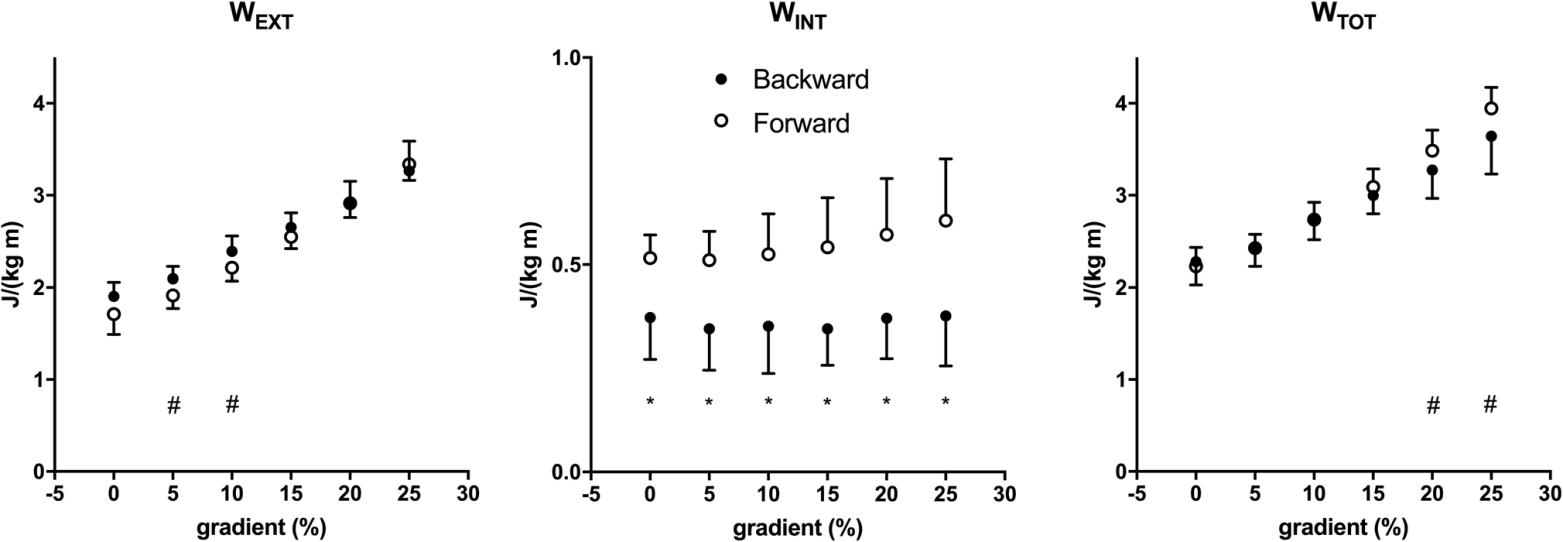
The mechanical external (*W*_EXT_), internal (*W*_INT_) and total (*W*_TOT_) work (J kg^−1^ m^−1^) as a function of gradient (%) is represented in backward (black circles) and forward (white circles) running. Statistical difference between backward and forward running: #*p* < 0.05; *p < 0.01. Data are mean ± SD

**Fig. 4:**
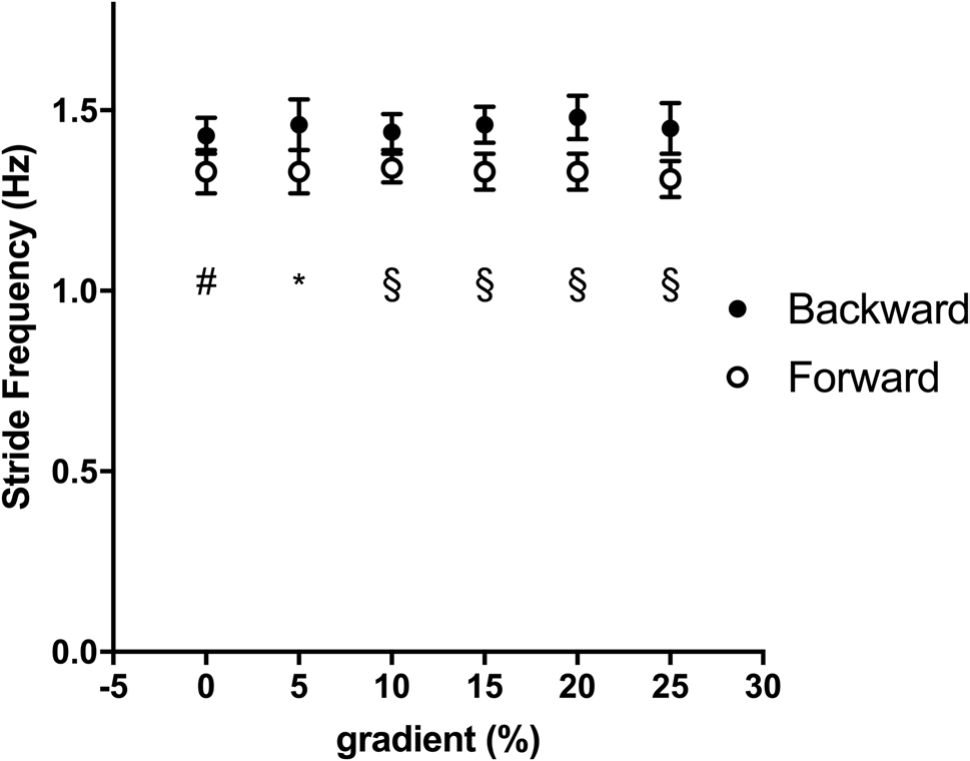
Stride frequency (Hz) as a function of gradient (%). Black circles represent backward running, and white circles represent forward running. Statistical difference between backward and forward running: #*p* < 0.05; *p < 0.01; §*p* < 0.001. Data are mean ± SD

The mechanical *W*_EXT_, *W*_INT_, and *W*_TOT_ of backward running in all gradient conditions are plotted as a function of speed in Fig. 2. *W*_EXT_ was the major determinant of *W*_TOT_ and decreased with speed, but increased with gradient. *W*_INT_ was almost gradient independent due to the decrease of speed. In Fig. 3, the mechanical parameters of backward and forward running are shown at each slope. Data were collected and presented at these identical gradients (0, + 5, + 10, + 15, + 20 and + 25%), however, at different speeds: 1.67, 1.53, 1.39, 1.25, 1.11, 0.97 m s^−1^ for backward running and 2.78, 2.50, 2.22, 1.94, 1.67, 1.39 m s^−1^ for forward running. *W*_EXT_ was greater in backward running from 0 to 10%, whereas W_INT_ was significantly lower in backward running at all gradients (*p* < 0.01) and *W*_TOT_ turned to be greater in forward running only at maximal gradients (20–25%, *p* < 0.05) (Fig. 3). Stride frequency (SF, Hz, Fig. 4) was statistically higher in backward than forward running at all slopes (*p* < 0.01).

**Fig. 5:**
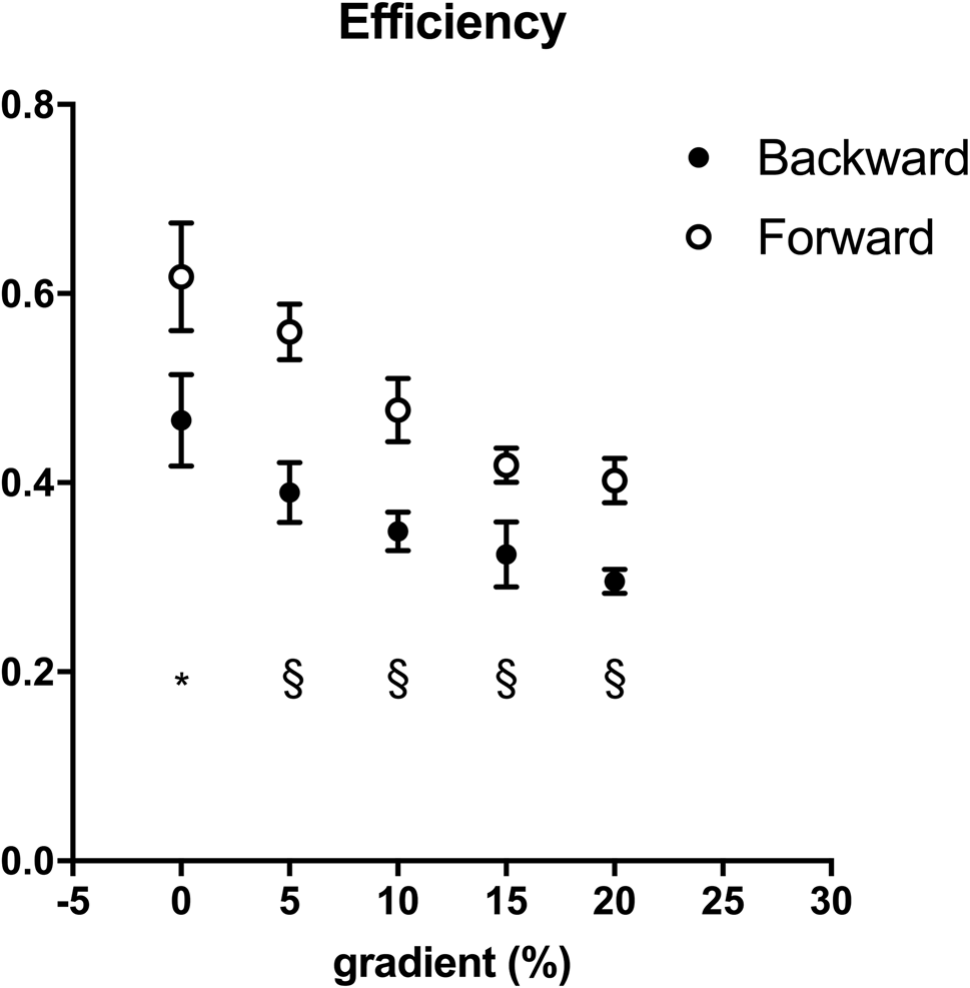
Running efficiency, calculated as the ratio between total mechanical work (*W*_TOT_, J kg^−1^ m^−1^) and metabolic cost (*C*, J kg^−1^ m^−1^), as a function of gradient (%) is represented in backward (black circles) and forward (white circles) running. Statistical difference between backward and forward running: *p < 0.01; §*p* < 0.001. Data are mean ± SD

Locomotion efficiency (Fig. 5) was greater in forward than backward running (*p* < 0.001) and decreased with gradient. Backward running reached values close to the muscular efficiency (0.25–0.30) at the steepest gradient where both metabolic and mechanical variable were measured.

**Fig. 6:**
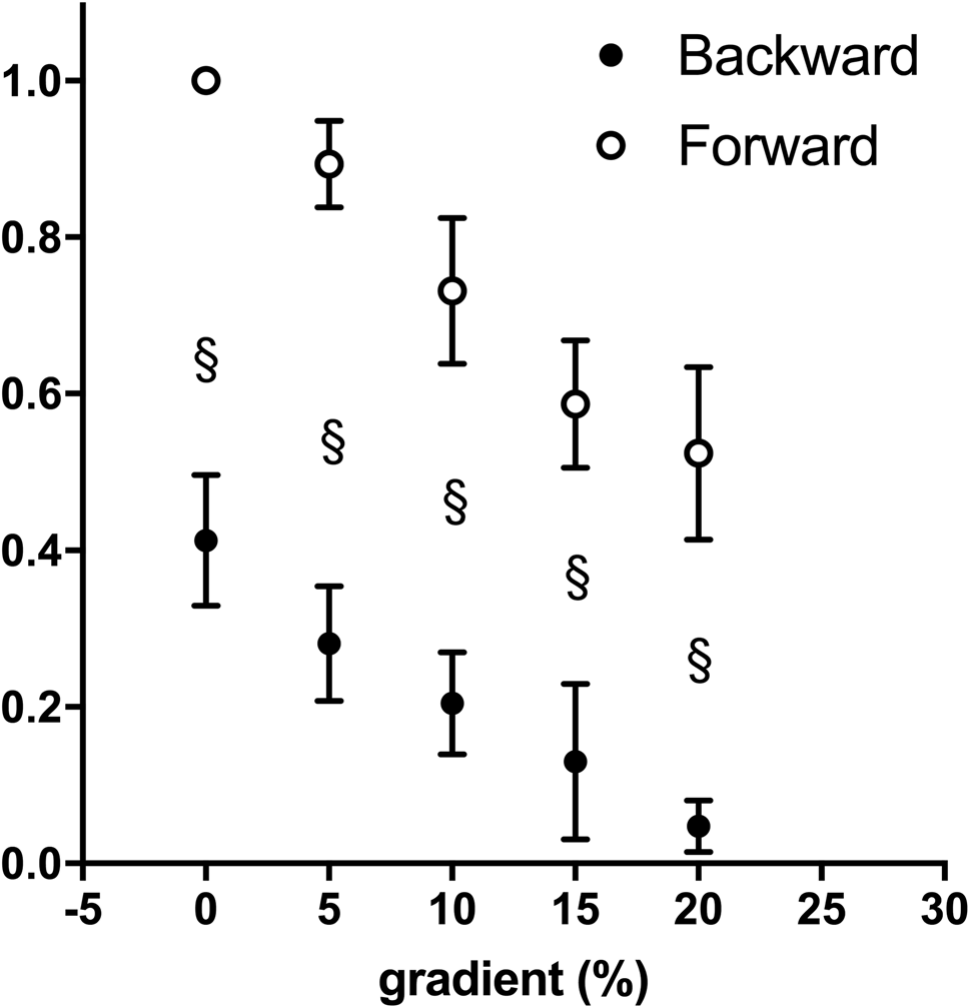
Estimated elastic energy contribution is represented as a function of gradient (%) in backward (black circles) and forward (white circles) running. The mean elastic energy of forward running on level is considered as 1 (see Material and methods for details), and all the other conditions are represented as submultiple. Statistical difference between backward and forward running: §*p* < 0.001. Data are mean ± SD

Estimated elastic energy contribution (Fig. 6) was higher in forward than backward running in all gradient conditions (*p* < 0.001) and decreased with gradient. Backward running approached no elastic energy contribution at the steepest gradient.

## Discussion

The metabolic cost of backward running was higher than forward running in all the investigated gradients, whereas the total mechanical work was similar in the two gaits at all gradients. Thus, the lower locomotion efficiency of backward than forward running (also on gradient) seems to be explained by the lower elastic energy contribution that does not assist muscles in performing mechanical work, which is carried out with a higher metabolic cost.

The metabolic cost of backward running was already shown to be higher than forward running on level over a range of speeds (Flynn et al. 1994; Wright and Weyand 2001) and the percentage difference is close to that reported in the present study. The novelty of this work consists in extending the previous knowledge also to gradients, where we found that the difference in metabolic cost was almost constant between the two running modalities at the different slopes, with a similar increase among gradients (Fig. 1). This behaviour differs from walking, since Minetti and Ardigò (2001) reported a decrease in delta cost between forward and backward walking on gradient, down to a + 5–8% difference at gradients steeper than 15%. They ascribed this decrease in delta cost to the impairment of the pendulum-like energy-saving mechanism of forward walking (energy recovery decreased in parallel with the metabolic cost) on gradient. Running does not rely on this mechanism, therefore a direct comparison cannot be performed; we will discuss later the energy-saving mechanism of running and its implication on the metabolic cost. The high metabolic power required for running backward forced us to test different speeds in the two running modalities, and to decrease speed (in both modalities) by increasing the gradient. The metabolic cost of forward running is speed independent on level and on gradient (Margaria 1938; Margaria et al. 1963; Minetti et al. 2002). Backward running C showed the speed independency on level (Wright and Weyand 2001), and here we extended this speed independency also on gradient [in the tested range of speeds (1.11–1.67 m s^−1^) and gradients (+ 10%, + 15%)]; thus, this speed difference between running modalities should not affect our metabolic conclusion.

The mechanical work values of backward running on level of present investigation showed the same pattern as in Cavagna et al. (2011) values (Fig. 2), whereas no data have been previously reported for backward running on gradient. *W*_EXT_ decreased with running speed, *W*_INT_ increased by increasing speed, but its contribution was small, and then *W*_TOT_ decreased in the investigated range of speeds at all gradients. The mechanical work data for forward running (Fig. 3) revealed similar trend compared with Minetti et al. (1994) values up to + 15%, which was the steepest gradient analysed in that study, whereas data on steeper slopes are not reported in the literature. At the two steepest gradients (+ 20 and + 25%), forward running *W*_EXT_ increased with the same trend as the previous gradients (Fig. 3). However, *W*_INT_ that was gradient independent until + 15% (present data and Minetti et al. 1994) showed a tendency to increase probably due to an increased duty factor and more extended limbs that increased the inertia during the swing (thus the compound factor *q* of the predictive equation for *W*_INT_ (Minetti 1998) is increased). This *W*_INT_ tendency to increase at the steepest gradients is similar to data reported by Nardello et al. (2011). A similar behaviour in the increase of *W*_INT_ and *q* factor has been reported at the beginning of the acceleration phase in sprint running (Pavei et al. 2019) and reinforces the idea that the mechanics of constant speed uphill running can be assimilated to running acceleration (di Prampero et al. 2005; Minetti and Pavei 2018). When comparing forward and backward running, albeit not at the same speed, on the different slopes the same trend in *W*_EXT_ and *W*_TOT_ was found, with *W*_EXT_ that increased linearly with gradient (on level *W*_EXT_ is higher in backward running, as reported by Cavagna et al. (2011)) and was the main determinant of *W*_TOT_. *W*_INT_ was slope independent in backward running, but showed a tendency to increase in forward running, which caused a higher *W*_TOT_ in forward than backward running at the steepest gradients. Stride frequency was higher in backward than forward running at all slopes (Fig. 4). On level, a higher stride frequency in backward compared with forward running at paired speed was already reported (Threlkeld et al. 1989; Flynn et al. 1994; Wright and Weyand 2001; Cavagna et al. 2011, 2012). Our results on level showed that speed (1.39–2.22 m s^−1^ range) was increased with a constant stride frequency and an increased stride length, similar to the results of Cavagna et al. (2012). The higher stride frequency would increase *W*_INT_, but we found higher values in forward than backward running. Other kinematics parameters concur in the computation of *W*_INT_: duty factor, defined as the fraction of foot contact within the stride duration, mean velocity and a compound *q* factor that accounts for the limb mass and spatial configuration during the stride (Minetti 1998). When analysing the differences of each *W*_INT_ component between backward and forward running on gradients, we found the already mentioned increase in stride frequency (+ 9%), an increase in duty factor (+ 27%), together with a decrease in velocity (− 36%) and *q* (− 32%), which led to a decreased *W*_INT_ (− 35%) in backward running.

**Fig. 7:**
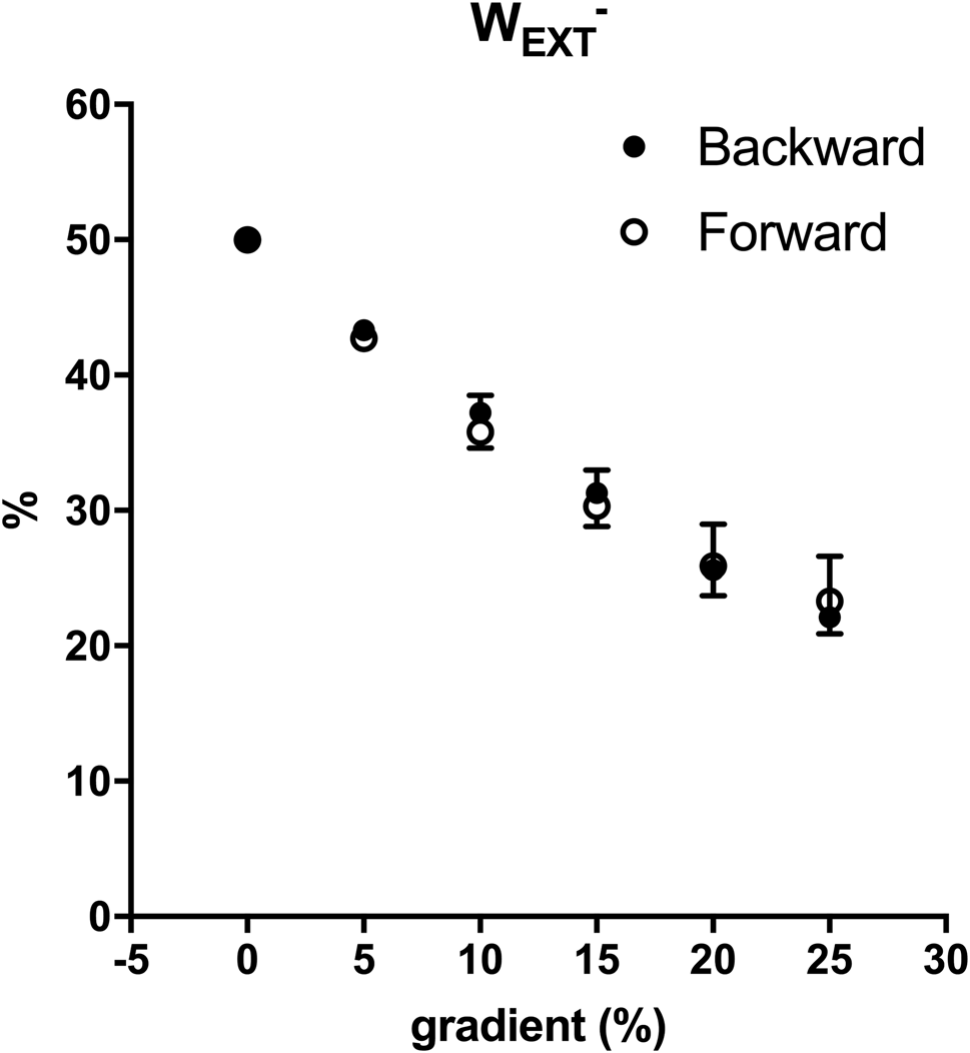
Negative external work (*W*_EXT_^−^) as a percentage of ‘comprehensive’ external mechanical work (= (*W*_EXT_) + (*W*_EXT_^−^)) is represented as a function of gradient (%). Black circles represent backward running, White circles represent forward running. Data are mean ± SD

Running has been classically represented as a bouncing ball (Cavagna et al. 1964) or a spring mass model (Blickhan 1989), where the lowering trajectory of the BCoM during the first half of the contact time compresses the spring (or deforms the ball) that can store elastic energy, which is then released to assist muscles while lifting and accelerating BCoM for the next step. Thanks to this elastic recoil of the muscle–tendon structures, running efficiency values are higher than the muscle efficiency (25–30%) and it is also termed ‘apparent efficiency’. In the present study, forward running apparent efficiency on level was ~ 60%, in line with the literature (Cavagna and Kaneko 1977), and decreased with increasing gradient, ~ 40% at + 20%, losing most of the ‘apparent’ part (Fig. 5). This is in accordance with and expand the results of Minetti et al. (1994). Apparent efficiency of backward running decreased similarly to forward running, but with about − 20% value in the slope range from level to + 20% (Fig. 5). These results showed that the energy-saving mechanism of running (the storage and release of elastic energy) is impaired on gradient. One explanation can be found by looking at the trajectory of the BCoM and the fraction of positive (*W*_EXT_) and negative external work (*W*_EXT_^−^) (Fig. 7). On level, positive (*W*_EXT_) and negative (*W*_EXT_^−^) external work equally contributes to the ‘comprehensive’ external mechanical work (= (*W*_EXT_) + (*W*_EXT_^−^)). By moving uphill, *W*_EXT_^−^ reduced its contribution as the BCoM trajectory became more ascending (as an effect of the slope) than descending (Minetti et al. 1994). Since the spring is compressed, and elastic energy is stored, during the lowering part of the trajectory, and this part is smaller by increasing gradient, less elastic energy can be stored. The muscles had then to perform the positive work to lift the BCoM, which increases with slopes, with less assistance from tendons; this required more metabolic energy that increased *C* (which is the denominator of the efficiency equation) and the efficiency decreased (Fig. 5). Since muscles are required to perform more work, a higher sEMG activity can be expected in backward than forward running; we did not assess sEMG, but higher activity was found when running backward on level (Flynn and Soutas-Little 1993, 1995; Sterzing et al. 2016). The partitioning between positive and negative external work was similar between the two running modalities (Fig. 7), highlighting the same behaviour of the BCoM trajectory on gradient. The estimated elastic energy contribution showed the same decreasing tendency with gradient of efficiency (Fig. 6), reinforcing the aforementioned idea that the energy-saving mechanism is impaired. Backward running values were always lower than forward running, and while at the steepest gradient forward running maintained some kind of elastic contributions, backward running relied only on muscle capability to perform work and power (Fig. 7). The mechanical inefficacy of backward running was already described by Cavagna et al. (2011, 2012) with the reversed landing take-off asymmetry, which resulted in a greater muscle activation during positive work and a lower ability to store and release elastic energy. These mechanical premises for inefficiency were tested here (since Cavagna et al. did not measure metabolic cost), confirmed in their original theory (elastic energy) and extended to the gradient, where we already knew that forward running energy saving was impaired (Minetti et al. 1994). Backward running with a reversed use of the lever system of the limbs that already impaired the efficiency on level showed the same impairment of forward running on gradient. However, starting from a lower level of ‘apparent efficiency’, at the steepest gradient backward running reached values of the ‘pure’ muscular efficiency, very likely with no elastic component.

Backward running is performed also in various sport activities, e.g. in soccer it has been reported to be as frequent as high-speed running (Mohr et al. 2003). However, up to now, backward running bouts are only counted (frequency of occurrence) and/or considered for their duration. The ‘Equivalent Slope’ concept has been an ingenious idea to infer the metabolic cost of running acceleration (di Prampero et al. 2005) from the metabolic cost of the steady-state uphill running (Minetti et al. 2002). With the present metabolic cost data of backward running on gradient (Fig. 1), we can calculate the metabolic cost of backward running over a range of 0–2 m s^−2^ acceleration (*C*_BA_, J kg^−1^ m^−1^). However, since the metabolic cost increased linearly with gradient in backward running (as occurred in forward running), we can expect that the proposed equation can be used over a wider range of accelerations. Rearranging the Minetti and Pavei (2018) equation for the metabolic cost in forward running acceleration with present data of backward running *C* on gradient, the cost of backward running acceleration can be computed as:

$$C_{{{\text{BA}}}} = \left( {a_{b}^{2} + 96.2} \right)^{0.5} \times \left( {3.14a_{b} + 4.9} \right),$$

where *a*_*b*_ is the absolute backward acceleration (a positive value, e.g. + 1.5 m s^−2^, even if it is performed backward, because the negative value is usually given to deceleration).

With this new equation, the metabolic power (= instantaneous *C*_BA_ $\times$ instantaneous speed) of backward acceleration can be computed, with the acceleration and speed values obtained from any GPS system, and added to the metabolic power for forward running acceleration and deceleration (Minetti and Pavei 2018) to obtain a more precise estimate of the metabolic power during different types of sports and activities.

## Conclusions

The metabolic cost of backward running on level and uphill gradient is higher than for forward running, with a similar difference between the two running modalities. This higher cost was not determined by an increased mechanical work; thus, the locomotion efficiency was lower in backward than forward running. When analysing the trajectory of the body centre of mass, the two running modalities showed a similar impairment in the spring mass model behaviour; however, backward running relied less on the elastic energy. With less elastic contribution, the muscles have to perform ‘alone’ the work to lift and accelerate BCoM with a higher metabolic demand. With the metabolic cost of backward running on gradient, and the concept of equivalent slope, the new equation for the metabolic cost of backward running acceleration was computed. The metabolic power of backward acceleration can be now calculated and integrated with the well-known equations for forward running acceleration and deceleration to obtain a more precise estimate of the metabolic demand of the sport activities.

## Abbreviations

*C*: Metabolic cost
*C*_BA_: Metabolic cost of backward running acceleration
BCoM: Body centre of mass
PE: Potential energy of BCoM
KE: Kinetic energy of BCoM
TE: Total energy of BCoM
*W*_EXT_: Positive external work
*W*_INT_: Positive internal work
*W*_TOT_: Total work
*W*_EXT_^−^: Negative external work
